# Early imaging biomarkers of lung cancer, COPD and coronary artery disease in the general population: rationale and design of the ImaLife (Imaging in Lifelines) Study

**DOI:** 10.1007/s10654-019-00519-0

**Published:** 2019-04-23

**Authors:** Congying Xia, Mieneke Rook, Gert Jan Pelgrim, Grigory Sidorenkov, Hendrik J. Wisselink, Jurjen N. van Bolhuis, Peter M. A. van Ooijen, Jiapan Guo, Matthijs Oudkerk, Harry Groen, Maarten van den Berge, Pim van der Harst, Hildebrand Dijkstra, Marleen Vonder, Marjolein A. Heuvelmans, Monique D. Dorrius, Peter Paul De Deyn, Geertruida H. de Bock, Aafje Dotinga, Rozemarijn Vliegenthart

**Affiliations:** 1 Department of Radiology, University of Groningen, University Medical Center Groningen, Hanzeplein 1, 9713 GZ Groningen, The Netherlands; 2 Martini Hospital Groningen, Groningen, The Netherlands; 3 Department of Epidemiology, University of Groningen, University Medical Center Groningen, Groningen, The Netherlands; 4 Lifelines Cohort Study, Groningen, The Netherlands; 5 Department of Radiation Therapy, University of Groningen, University Medical Center Groningen, Groningen, The Netherlands; 6 iDNA B.V, Groningen, The Netherlands; 7 University of Groningen, Groningen, The Netherlands; 8 Department of Pulmonary Diseases, University of Groningen, University Medical Center Groningen, Groningen, The Netherlands; 9 Department of Cardiology, University of Groningen, University Medical Center Groningen, Groningen, The Netherlands; 10 Department of Neurology, University of Groningen, University Medical Center Groningen, Alzheimer Research Center, Groningen, The Netherlands

**Keywords:** Cardiovascular disease, Chronic obstructive pulmonary disease, General population, Imaging biomarkers, Low-dose computed tomography, Lung cancer

## Abstract

Lung cancer, chronic obstructive pulmonary disease (COPD), and coronary artery disease (CAD) are expected to cause most deaths by 2050. State-of-the-art computed tomography (CT) allows early detection of lung cancer and simultaneous evaluation of imaging biomarkers for the early stages of COPD, based on pulmonary density and bronchial wall thickness, and of CAD, based on the coronary artery calcium score (CACS), at low radiation dose. To determine cut-off values for positive tests for elevated risk and presence of disease is one of the major tasks before considering implementation of CT screening in a general population. The ImaLife (Imaging in Lifelines) study, embedded in the Lifelines study, is designed to establish the reference values of the imaging biomarkers for the big three diseases in a well-defined general population aged 45 years and older. In total, 12,000 participants will undergo CACS and chest acquisitions with latest CT technology. The estimated percentage of individuals with lung nodules needing further workup is around 1–2%. Given the around 10% prevalence of COPD and CAD in the general population, the expected number of COPD and CAD is around 1000 each. So far, nearly 4000 participants have been included. The ImaLife study will allow differentiation between normal aging of the pulmonary and cardiovascular system and early stages of the big three diseases based on low-dose CT imaging. This information can be finally integrated into personalized precision health strategies in the general population.

## Rationale

Lung cancer, chronic obstructive pulmonary disease (COPD) and coronary artery disease (CAD) are in the top ten global causes of death [1]. In this top ten, over three-quarters of individuals dying of non-communicable diseases, succumb to one of these three diseases, sometimes called the ‘big-3’ (B3).

Early detection of B3 diseases by low-dose computed tomography (CT) could provide opportunities for early treatment and possibly reduce mortality in the overall population. Lung cancer screening using low-dose chest CT is now recommended to detect early cases of lung cancer [2]. Quantification of CT imaging biomarkers such as lung density and bronchial wall thickness may allow early detection of COPD [3, 4]. As low-dose chest CT for lung cancer screening inherently includes the heart in its field of view, this has the potential for the evaluation of CAD as well. Efforts are underway to develop a CT scan protocol that will allow accurate evaluation of B3 imaging biomarkers with one image acquisition [5].

Although B3 diseases share certain risk factors (aging and smoking) and mechanisms (systematic inflammation and endothelial dysfunction), there are also important differences in risk factors between lung and heart diseases that can affect screening recommendations. Current lung cancer screening recommendations focus on high-risk, long-term smokers. For COPD and CAD, at this moment no at-risk groups are defined that may benefit from screening. Excellent large-scale imaging studies have been performed aimed at investigating one of the B3 diseases (see Table 1). The lung cancer imaging studies have been interventional screening studies in a high-risk population, while studies on CAD and COPD have generally been observational in design, and for CAD often included a more general population. A smaller number of studies included evaluation of a second B3 disease, often based on the same CT scan (which is suboptimal for evaluation of the other B3 disease), or by performing an additional dedicated CT scan, usually in a subset. There is so far no population imaging study that is focused on obtaining the B3 imaging biomarkers in a comprehensive, integrated approach with an optimized CT scan protocol. In view of the different risk factors for individual B3 diseases, it is not unreasonable to contemplate the possibility of B3 screening in the general adult population, or in an optimized selection of the population based on (combinations of) risk factors for premature development of B3 diseases.

**Table 1:** Large-scale studies with evaluation of at least two of the big three diseases based on (semi-)quantitative imaging biomarkers

Study	Population description	Overall sample size	Age (years)	Primarily aimed at			Ancillary studies aimed at (sample size)		
				Lung cancer	COPD	CVD	Lung cancer	COPD	CVD
SCAPIS [46]	General population	30,000	50–64		‡	√			
NELSON [4, 9, 37]	Heavy (ex-)smokers	15,822	50–75	○				‡ (1,140)	√ (3,111)
NLST [8, 38, 47]	Heavy (ex-)smokers	53,454	55–74	×				‡ (558)	√ (1,575)
MILD [36, 39]	Heavy (ex-)smokers	4099	≥ 49	○				† (1,159)	√ (1,159)
I-ELCAP [34, 40, 48]	At risk for lung cancer (i.e., heavy smoker, occupational exposure)	31,567	≥ 40	×				† (9,047)	√ (8,782)
ITALUNG [49, 50]	Heavy (ex-)smokers	3206	55–69	×				† (266)	
DLCST [35, 41]	Heavy (ex-)smokers	4104	50–70	×					√ (1,945)
PLuSS [51, 52]	Heavy (ex-)smokers	3642	50–79	×				‡ (234)	
ECLIPSE [42, 53]	Patients with GOLD stage II–IV COPD	2161	40–75		†				√ (942)
COPDGene [43, 54]	Current or former smokers with at least 10 pack-years of exposure to smoking	10,000 (planning)	45–80		‡				√ (1,875)
MESA [16, 44]	Free of apparent cardiovascular disease	6814	45–84			√		‡ (> 3,000)	
Framingham heart study [55, 56]	Community-based cohort in Framingham	3529 (underwent scanning)	Men ≥ 35; women ≥ 40			√		‡ (nearly 3000)	

√, coronary calcium scoring as a imaging biomarker for CVD; ‡, Lung density (i.e., emphysema, air trapping) and airway wall thickness as imaging biomarkers for COPD; †, Lung density based imaging biomarker for emphysema; ○, volume (volume doubling time) of lung nodules as imaging biomarkers for early stage of lung cancer; × , diameter of lung nodules as imaging biomarkers for early stage of lung cancer

*COPD* chronic obstructive pulmonary disease, *CVD* cardiovascular disease, *CACS* coronary artery calcium score, *SCAPIS* Swedish CArdioPulmonarybioImage Study, *NELSON* Dutch-Belgian Lung Cancer Screening trial, *NLST* National lung cancer screening trial, *MILD* Multi-centric Italian Lung Detection, *I*-*ELCAP* international early lung cancer action program, *DLST* Danish lung cancer screening trial, *PLuSS* Pittsburgh Lung Screening Study, *ECLIPSE* Evaluation of COPD Longitudinally to Identify Predictive Surrogate End-points, *GOLD* global initiative for chronic obstructive lung disease, *MESA* Multi-Ethnic Study of Atherosclerosis

ImaLife is the first large-scale imaging study focusing on combined evaluation of early imaging biomarkers of the B3. In the ImaLife study, a low-dose CT scan is acquired with dedicated protocol for cardiac and lung imaging biomarkers. With help of these data, we will determine reference values of early imaging biomarkers of the B3 in the general adult population. This will allow differentiation between normal ageing and premature development of subclinical B3 diseases by age and gender. Instead of treating these as three independent diseases, a B3 concept to manage these degenerative diseases holistically might be more effective [6]. In ImaLife, inter-relatedness between early stages of B3 diseases will be investigated. Furthermore, these data will help to define an at-risk population that could benefit from screening for B3 diseases.

## Objectives

The primary objective of ImaLife study is to establish CT reference values of quantitative imaging markers for the B3 diseases in the general population aged 45 years and older, namely lung nodules for lung cancer, lung density and bronchial wall thickness for COPD, and CACS for CAD. As the ImaLife study is embedded in the Lifelines cohort, a large prospective cohort with extensive data collection in the north of the Netherlands [7], the study will also allow the (1) analysis of correlations of imaging biomarkers to lifestyle factors, demographic characteristics, environmental factors, and other clinical biomarkers including conventional laboratory biomarkers, metabolic biomarkers and so forth, (2) investigation of the inter-relationship between early stages of B3, (3) identification of at-risk subgroups in the population for premature development of the B3, (4) validation of the value of early imaging biomarkers for predicting clinical stages of the B3 diseases, and (5) investigation of imaging biomarkers for other degenerative diseases such as osteoporosis.

## Methods

### Current state-of-the-art

The National Lung Screening Trial (NLST) showed the benefit of low-dose CT to reduce lung cancer mortality in at-risk individuals [8]. However, there was a high false-positive rate due to the single measurement of a nodule diameter for determining screen result. The key to reduce false-positive rates may be found in using semi-automated volumetric nodule quantification and calculating volume doubling time (VDT) of indeterminate lung nodules [2]. The Dutch-Belgian lung cancer screening trial (NELSON) provided evidence that volume-based CT screening is an effective method of lung cancer screening, with much lower false positives [9, 10]. Reference values of nodule volume and VDT are important, especially when aiming to prevent unnecessary follow-up CT scans and unnecessary fear of cancer. While lung cancer screening studies focused on high-risk long-term smokers, little is known about the prevalence and size and growth of lung nodules in a general population.

The coronary artery calcium score (CACS) is the strongest non-invasive predictor for coronary events [11–16], and its impact as screening tool is currently under investigation [17]. Previously, the Heinz Nixdorf Recall (HNR) study [15] and the Multi-Ethnic Study of Atherosclerosis (MESA) [16] provided the distribution of CACS in unselected adult populations without clinical CVD in Germany, and USA respectively. The Rotterdam study evaluated the CACS in the older population [18]. Apart from MESA, these studies only focused on CAD, without relating CACS to other B3 diseases.

Early stages of COPD can be quantified by measuring emphysema, bronchial wall thickness and air trapping on chest CT scans [19]. Structural lung abnormalities on CT were found to increase the risk of lung cancer and mortality [20–22]. Presence and severity of COPD imaging biomarkers in the general population are largely unknown. In a subset of MESA, the cardiac CT scan was used to determine some estimate of COPD measures, however, the higher lung fields, where emphysema typically starts, were not imaged.

### Lifelines

The Lifelines study is a longitudinal cohort study and biobank in a large three-generation population in the northern part of the Netherlands, comprising over 167,000 participants [7, 23]. The Lifelines cohort study has been established in 2006. Baseline assessments were completed in 2013, after which second-round assessments have been started and completed in 2017. The third-round assessments will start in 2019. Participants are invited to complete a physical examination with lab testing once every assessment round, and questionnaires once (on average) every 1.5 years. Not only medical information but also environmental data are collected to investigate the complex interaction between possible risk factors, chronic diseases, and healthy aging. More details about specific variables that collected in the Lifelines biobank can be found at [www.lifelines.nl]. Lifelines has regular examination rounds, in which participants, amongst other exams, fill in questionnaires. The questionnaire includes questions about health status, and the occurrence and prevalence of disease such as lung cancer or CAD. This is the primary way in which information regarding incidence of B3 diseases is obtained within Lifelines [7]. Also, Lifelines is working to create a link with GP data, in order to obtain clinical diagnoses of the participants. From the Lifelines cohort, 12,000 participants aged 45 years and above are invited for the ImaLife substudy (expected age range 45–94 years, and at least 50% women).

### ImaLife study population and recruitment strategies

**Fig. 1:**
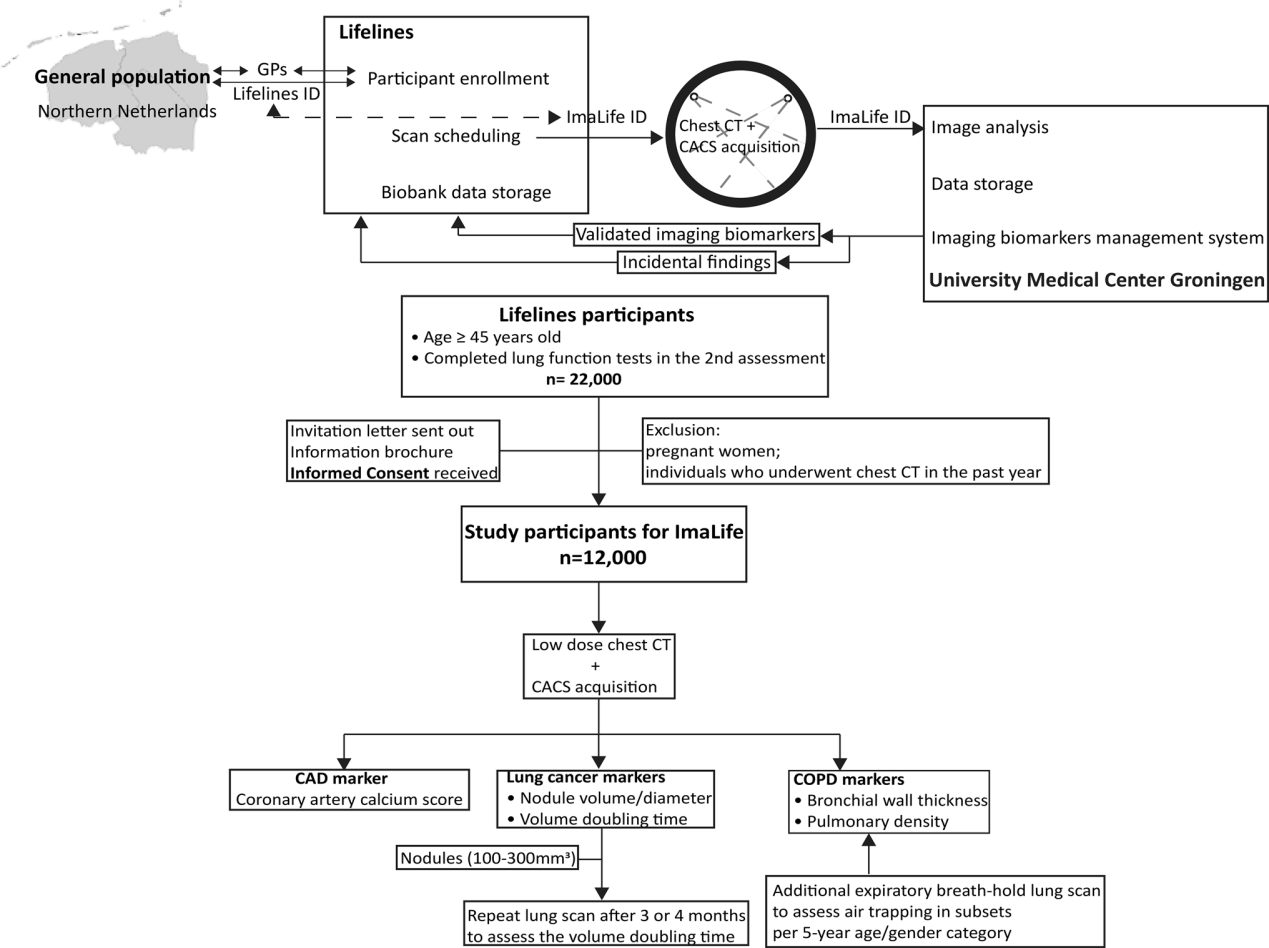
Overview of the ImaLife study. *CT* computed tomography, *COPD* chronic obstructive pulmonary disease, *CAD* coronary artery disease, *GPs* general practitioners, *ID* identification number

The ImaLife substudy is an imaging study with state-of-the-art CT scanning procedures embedded in Lifelines. For an overview of the study, see Fig. 1. The ImaLife study includes Lifelines participants who completed the second round assessment of the Lifelines study, and as part of the second round assessment, completed a lung function test. The cohort invited for lung function testing was unselected (primarily based on scheduling of lung function test slots). However, there was a number of exclusion criteria for lung function testing, related to conditions in which intrathoracic pressure increase need to be prevented, namely: (1) known abdominal hernia (including inguinal, umbilical, incisional), rib fracture, aneurysm, or tuberculosis, (2) recent cardiac catheterization (within 2 weeks prior), (3) recent respiratory infection (within 3 weeks prior), (4) recent (within 6 weeks) surgery for abdominal hernia, ocular surgery, thoracic or abdominal surgery, (5) recent (within 6 weeks) pneumothorax or more than 2 times spontaneous pneumothorax, (6) recent (within 2–6 weeks) pulmonary embolism, (7) recent (within 2–6 weeks) myocardial infarction, (8) indication by general practitioner or medical specialist that no (heavy) exercise was to be performed. Also, participants who were unable to complete the lung function test due to dizziness or hyperventilation were excluded. In total, 32,113 Lifelines participants completed the lung function test (age range 18–94 years, 58.8% female). Of these, all Lifelines individuals aged 45 years and above are invited for the ImaLife study (n = 22,000).

A cut-off of age 45 years is chosen because early B3 stages, as well as positive imaging biomarkers for lung nodules, COPD and CAD are rare below 45 years [24–26]. This lower cut-off is similar to other large-scale imaging studies (see Table 1). Even though f.e. coronary calcium is less prevalent in women than in men, it is important to have the same lower age cut-off to be able to compare early B3 imaging biomarkers in terms of presence and extent for men and women at the same age.

Participants who are invited to participate in the ImaLife study receive an invitation letter, an information brochure about the ImaLife study including discussion of possible risks and benefits, an informed consent form and an availability form. Participants who return the signed informed consent will be included in the ImaLife study. We exclude pregnant women and individuals who underwent chest CT in the past year. The latter because of the probability of existing clinically potential life-limiting B3 disease. Subsequently, participants receive an appointment to undergo the CT scan from the Lifelines organization. With an expected positive response rate for the invitation 50–60%, the total number of participants for the ImaLife study will be around 12,000. For the primary objective, outcomes will be obtained for all participants, namely quantitative imaging biomarkers on lung nodules, lung density, bronchial wall thickness, and CACS. This allows determination of reference values of the imaging biomarkers of early stages the B3 in this general population by gender and 5 or 10-year age groups.

### Image acquisition and analysis

Study participants undergo a low dose chest CT examination with third-generation dual-source CT (SOMATOM Force, Siemens, Germany). First a CACS acquisition with electrocardiography (ECG) triggering is performed, followed by a low dose chest CT acquisition. The protocol of cardiac CT is based on the acquired experience of the ROBINSCA study [17], while the protocol of low-dose thoracic CT is modified from the low-dose CT protocol in the NELSON study [27]. Both are adapted to the latest generation dual-source CT system. In addition, a subset of 100 participants for each 5-year age/gender category (in total, about 1000 participants) will undergo an additional acquisition with end-expiratory breath hold, in order to determine normal values for expiratory lung density and air trapping. Details of the acquisition and reconstruction parameters are presented in Tables 2 and 3. Radiologic technologists who are trained in the low-dose CT scan protocol are responsible for CT scanning.

**Table 2:** CT scanning protocol for coronary artery calcium score and lung scan

Acquisition parameters	CT protocol	
	CACS	Lung
Scan mode	High pitch spiral	High pitch spiral
Pitch	3.2	3.0/2.5^a^
Tube voltage (kVp)	120	120
Tube current reference (ref mAs)	64	20
Rotation time (ms)	250	
Matrix	512 × 512	
ECG triggering	Yes, prospective at 60% of the R–R interval	No
Dose modulation	Care-kV off	Care-kV off
API	Inspiratory breath-hold	Inspiratory/expiratory breath-hold^b^
Direction	Craniocaudal	
Upper limit	Below carina	Above lung apex
Lower limit	Apex/bottom edge heart	Caudal edge lungs

^a^The pitch is set as 3.0 for lung scans with a field of view 350 mm for reconstruction, while the pitch will be modified to 2.5 with a field of view enlarged to 400 mm for reconstruction in participants with larger body sizes

^b^A subset of 100 participants per 5-year age/gender category will undergo an additional acquisition with end-expiratory breath hold

*CT* computed tomography, *CACS* coronary artery calcium score, *ECG* electrocardiography, *API* automated patient instruction

**Table 3:** CT reconstruction protocols for coronary artery calcium score and lung scan

Reconstruction parameters	CACS	Lung		
	Reconstruction	Reconstruction 1	Reconstruction 2	Reconstruction 3
Slice thickness (mm)	3.0	1.0	1.0	1.0
Slice increment (mm)	1.5	0.7	0.7	0.7
FOV (mm)	250	350 (400 if 350 too small)	350 (400 if 350 too small)	350 (400 if 350 too small)
Reconstruction kernel	Qr36 (Quantitative-medium sharp)	Bl57 (Qualitative-sharp)	QR59 (Quantitative-sharp)	Br40 (Qualitative-smooth)
Reconstruction method	FBP	FBP	FBP	FBP
Window	Mediastinum	Lung	Lung	Mediastinum
Window width (HU)	350	1600	1600	350
Window center (HU)	50	− 400	− 400	50

*CACS* coronary artery calcium score, *FOV* field of view, *FBP* filtered back projection, *HU* hounsfield units

Syngo.via software (version VB30) with MM Oncology application (Siemens Healthineers, Germany) will be used for lung analysis and volumetric quantification of lung nodules. If a non-calcified lung nodule is found (100–300 mm^3^), the participant is invited for a repeat low-dose CT scan of the lungs in inspiration after 3 to 4 months to study the persistence of nodules, and reference values of VDT in the general population. The VDT will be determined basing on the formula as previously reported [28].

Evaluation of COPD will be performed on low dose chest CT scans with inspiratory and expiratory breath hold Bronchial wall thickness and pulmonary volume and density will be analyzed using semi-automated software (Pulmo3D, Siemens Healthineers, and others).

Coronary calcification will be evaluated using semi-automated CACS software (CaSc, Siemens Healthineers, Germany). Agatston score, volume score and mass score will be archived, overall and per artery. Although coronary calcification is a predictor for cardiovascular disease [11, 13, 14], there is no clinical evidence that early intervention in case of higher burden of coronary calcification can reduce cardiovascular risk or improve clinical outcomes so far. Therefore, in view of ethical considerations in the context of ImaLife being an observational population study, the result of CACS will not be reported to study participants and GPs.

Reading of the scans is performed by and/or under the supervision of radiologists (6–12 years of experience in cardiothoracic imaging). Researchers with a background in (technical) medicine perform the measurements of lung nodule volumes, COPD imaging biomarkers, and calcium scoring, after adequate training (educational dataset of at least 50 cases for lung nodules and 50 cases for calcium scoring, with feedback on trainee evaluations).

### Notification of incidental findings

ImaLife is in principle an observational study. In accordance with the observational nature of the study, information on early imaging biomarkers of COPD or coronary calcium, and lung nodules with small volume, is not reported to the participants. B3 imaging biomarkers are considered early imaging biomarkers of subclinical disease that may or may not lead to clinical expression in the future. Only an incidentally detected, potentially life-shortening and thus clinically relevant finding will be reported to the participant and his/her GP. This finding may require further management if it was not previously known. In case the radiologist notices a potentially clinically relevant finding while reviewing the lung scan, he/she will report this in the digital notification system to Lifelines. The findings that are reported if incidentally detected, are listed in Table 4. In such cases, the GP will be the first to receive the information regarding the potentially clinically relevant finding. Results regarding incidental findings are in principle available within 10 working days after the CT scan and sent to the GP. Participants will receive a letter within 10 working days after the GP receives the result. The participant is advised to contact the GP for more information and referral to a medical specialist, if the incidental finding was not previously known. Taking the medical history into account, the GP will make the final decision to refer the participant to a medical specialist to further investigate the finding. The medical specialist can request a DVD with the (anonymized) CT scan. One of the incidental findings that is reported to participant and GP, is a lung nodule of at least 300 mm^3^, according to published recommendations [2], as it cannot be ruled out that a nodule with this volume is an early stage of pulmonary malignancy that is assumed to inevitably result in clinical expression. Incidental findings without evident potential clinical relevance will not be reported in view of ethical considerations in this non-screening, observational study in the general population.

**Table 4:** Incidental findings on the low-dose CT scan that are and that are not reported to participant and GP, if accidentally detected

	Reported	Not reported
Chest	Aortic aneurysm ≥ 50 mm	Valve calcification (aortic, mitral, etc.)
	Calcified pleural plaques ≥ 1 cm thickness	Annulus calcification
	Pleural fluid ≥ 2 cm thickness	Pericardial abnormalities (thickening, calcification, etc.)
	Lung nodule with size ≥ 300 mm^3^ (0.8 cm) or fast growing (VDT < 400 days) if 100–300 mm^3^ (0.6-0.8 cm)	Hiatal hernia
	Identifiable thoracic mass (> 3 cm)	
Abdomen	Very large liver cyst(s) (> 10 cm)	Small to medium size liver cyst(s)
	Identifiable abdominal mass (> 3 cm)	

*VDT* volume doubling time

### Radiation dose management

With the use of third-generation dual-source CT and modified scan protocols, the effective radiation dose for participants undergoing a baseline ImaLife CT scan will be between 0.6 and 1.8 mSv, depending on weight and length. Additionally, the subset of participants who undergo an expiratory breath hold scan, will receive an extra 0.1 to 1.3 mSv. In case of a follow-up scan (nodule 100–300 mm^3^, expected in 5–10% of participants), the repeat thorax CT scan in three to four months will result in an additional 0.8 mSv on average. To put these estimates into perspective, the natural background radiation dose in the Netherland is about 2.5 mSv per year [29]. These dose ranges fit within the radiation dose limits for population imaging as described by the Dutch Health Council [30]. The CT system will provide a dose warning when the projected radiation dose for the CT scan will exceed the upper dose limit based on dose-length product. The overall radiation dose will be checked at regular intervals in order to check the actual radiation dose in the scanned population.

### Data storage and management

Participants are invited by Lifelines and are linked to a new ImaLife participant identification number (ID) that is unrelated to their Lifelines participant ID. The CT scanning and scan evaluation is performed using this specific ImaLife participant ID. Only Lifelines has the key to link pseudonymous imaging biomarkers to the Lifelines database. By this method, the researchers involved in ImaLife who are performing the data evaluation have no link whatsoever to the real identity of the participant. This identity can only be retrieved by Lifelines, which is why communication to the participants or their GP is done by Lifelines. In this setup, Lifelines is acting as a Trusted Third Party thus achieving optimal protection of the privacy of the participants. Pseudonymous scans will be uploaded to a secure virtual research workspace which allows access to imaging software for analyses, connection with research Picture Archiving and Communication System (PACS), and linkage to data storage. Image analyses will be conducted by the trained researchers and radiologists via the ENACT workspace. Registration of all measurement results is made in a dedicated ImaLife data management system. Only validated imaging markers derived from the quantitative image analyses will be added to the Lifelines database.

### Statistical considerations

The percentage of women in Lifelines is relatively high (58.5% versus 50.7% in the entire population of the Northern Netherlands) [23]. In the Lifelines cohort, there is a sufficient prevalence of ever smoking and (other) cardiovascular risk factors, with more than 70% of participants having at least one traditional cardiovascular risk factor [31].

Within the B3, COPD and CAD are most common with 10% prevalence including early stages in the adult population. The expected number of individuals with early stages of COPD and CAD is at least 1000 each, allowing detailed analyses of the research questions. The study is calculated to have more than 95% power to identify the relationship between risk factors with a prevalence of at least 10% in the ImaLife cohort and presence of coronary calcium with an odds ratio of 1.3 or greater, and presence of COPD with an odds ratio of 1.5 or greater, given an alpha error of 5%. It is expected that there will be approximately 900 CVD events and 300 COPD events occurring in a 5 year period, given the incidence rate of CVD and COPD of nearly 15 and 5 per 1000 per year, respectively [32, 33], allowing the analyses of the relationship between imaging biomarkers and outcomes. The prevalence of (early) lung cancer in the general population above 45 years is low, although lung nodules are common (about 50% in (ex-) smokers, based on the NELSON study, and 2.6% with a positive screen test [27]). The prevalence of current smoking in Lifelines is about 25%, which is lower compared to the NELSON study. We hereby estimate that there will be about 120–240 individuals (1–2%) with a positive test that need referral to a pulmonologist and further work-up. This is adequate to answer the research questions for the whole population, but insufficient to perform analyses in subgroups of gender, age, and environmental exposure.

### Ethical considerations and privacy issues

The ImaLife study was approved by the medical ethics committee of the University Medical Center Groningen, the Netherlands. All participants sign an informed consent form prior to the investigational procedures; informed consent forms are stored at the Lifelines organization. The ImaLife study was registered with the Dutch Central Committee on Research Involving Human Subjects (https://www.toetsingonline.nl, Identifier:NL58592.042.16). By implementing a twofold de-identification process decoupling the identity of the participant completely from the ImaLife ID, we are complying with the rules and regulations concerning the protection of privacy (General Data Protection Regulation—GDPR).

## Discussion

The ImaLife study will establish reference values of early imaging biomarkers for the B3 diseases, namely, lung nodules (volume and VDT), bronchial wall thickness, pulmonary density, and CACS in the general population in the northern part of the Netherlands aged 45 years and above. These reference values can be used in the future to determine premature, subclinical development of B3 diseases, and for modeling personalized prevention strategies in the general population.

To the best of our knowledge, the ImaLife study is the first population-based imaging study aiming to detect the early stages of the B3 diseases and to establish reference values for the B3 imaging biomarkers. Previous studies were primarily aimed at one or two of the B3 diseases, generally in a selected population (summarized in Table 1). For instance, lung cancer screening trials including NELSON, NLST, MILD, I-ELCAP, DLCST were originally designed to show whether screening of lung cancer in targeted-population with low-dose chest CT could reduce mortality [8, 27, 34–36]. But CACS based on the non-ECG-triggered chest CT was reviewed later as an ancillary study to investigate the predictive value of CACS for cardiovascular events, due to the visibility of the heart on regular chest CT [37–41]. The same goes for the ECLIPSE study and the COPDGene study which were primarily designed for COPD evaluation [42, 43]. Vice versa, the MESA study, which was originally established for subclinical cardiovascular disease and risk factors assessment, was expanded with a lung scan acquisition to test the endothelial hypothesis of COPD and emphysema [44]. Compared with these large population studies with imaging, the ImaLife study is originally aimed at the B3 diseases. It acquires scans in a combined, optimized approach for optimal biomarker evaluation, allowing an integrated cross-sectional analysis of correlation among the B3 imaging biomarkers. This can give a clue to what extent the (early stages of) B3 diseases are interconnected and which factors influence the premature development of one, two or three B3 diseases, using the rich database of the Lifelines cohort, which can help to identify an ‘at-risk’ subgroup in the population.

The ImaLife study has several strengths. First, it is a population-based observational study with a large sample size. Second, it is a comprehensive radiology study with state-of-the-art low-dose CT, standardized scan and evaluation protocols, dedicated software and sophisticated support from ICT technicians. Third, the ImaLife study is embedded in the prospective Lifelines cohort study. This allows the longitudinal investigation of the relationship between imaging biomarkers and clinical progression of the B3 diseases. In addition, the wealth of information in the Lifelines database, including environmental factors and microbiome, allows correlation with early imaging biomarkers in order to detect new factors influencing the development of B3 diseases. Not only conventionally quantitative imaging biomarkers are measured, but also new image features are extracted (f.e. using radiomics) allowing potentially more predictive information from the same CT scan. Also, early imaging biomarkers for other degenerative diseases, such as liver density for non-alcoholic fatty liver disease, bone density or vertebral fractures for osteoporosis, and muscle mass can be determined. These imaging biomarkers would help in predicting mortality. Lastly, following the ImaLife study design, a corollary study, NELCIN-B3, is being set up in the Chinese population in collaboration with Chinese academic hospitals. This allows the comparison of reference values in a Dutch and Chinese population.

Besides the clinical strengths, the ImaLife study will also contribute to the development of computer-aided diagnosis systems. With the rise of deep learning approaches in computer vision, artificial intelligence techniques are widely employed in different disciplines to assist domain experts in their daily practical work. The development of such a system, however, requires not only the implementation of the techniques but more importantly the feeding of sufficient amounts of labelled data for the supervised training of the system. From this point of view, the collection of scans from the ImaLife study and the corresponding evaluation by trained readers (including labelling of the data) will be beneficial and necessary for the development of such a system. In return, the computer-aided diagnosis system will further support the population-based screening study in the sense of ruling out normal aging cases, detecting early signs of B3 diseases and expectantly discovering new biomarkers for the diagnosis of certain diseases.

The weaknesses of the study also need to be addressed. Firstly, the potential for selection bias. Participants of the ImaLife study are recruited from the Lifelines cohort. The Lifelines cohort is a broadly representative sample of the general population of the northern Netherlands, however there is a potential source of selection bias regarding enrollment in the ImaLife study, as participants were derived from the (unselected) cohort that underwent lung function testing for which there was a number of exclusion criteria. Also, individuals below 45 years of age are not included, as the expected prevalence of early B3 stages and positive imaging biomarkers for lung nodules, COPD and CAD are rare below 45 years [24–26]. The participation rate for ImaLife CT scanning among healthier and younger individuals may be higher than the rate in older and less healthy individuals, which may lead to bias when the reference values of imaging markers derived from ImaLife are to be generalized to the larger population. Furthermore, the elderly participants who do participate in the ImaLife study may be less representative for their age groups, because of their robust health status. Additionally, a potentially lower response rate in the elderly may result in insufficient power when age-stratified analysis is conducted, and the power will be worse for the investigation of the relationships with rare risk factors. Another weakness is that CT scans for lung and for heart are currently performed as separate acquisitions, which results in slightly higher radiation dose. The total study related radiation dose will be between 0.6 and 1.8 mSv for these two acquisitions (with an additional 0.1 to 1.3 mSv in a subgroup undergoing expiratory CT). The risk of long-term health effects from radiation dose in this range is either too small to be observed or does not exist [45]. However, in the future, combined low-dose chest CT might possibly be used to gather the B3 imaging biomarkers in one scan. This approach needs, however, further validation in order to secure accurate imaging biomarker quantification.

## Conclusion

The ImaLife study, embedded in the Lifelines cohort, is a population-based study in the northern part of the Netherlands. Scan data acquisition started at the end of August 2017. So far, around 4000 participants have been enrolled. The enrollment is expected to be completed in the second half of 2020. Participants will be regularly followed up according to the Lifelines cohort procedures. The ImaLife study will yield reference values of early imaging biomarkers for the B3 diseases in the general population, and help integration of the B3 imaging biomarkers into personalized prevention strategies for healthy ageing.
